# Intensive care infection score: ICIS discriminates between infected and uninfected critically ill patients in routine intensive care unit practice

**DOI:** 10.1186/s40635-025-00767-3

**Published:** 2025-06-05

**Authors:** Emre Deniz, Stefanie Klatte, Nilgün Tekin-Bubenheim, Mathias Zimmermann

**Affiliations:** 1 Institute of Laboratory Medicine, DRK Kliniken Berlin Westend, Spandauer Damm 130, 14050 Berlin, Germany; 2 Medical Science Department, Sysmex Europe SE, Bornbarch 1, 22848 Norderstedt, Germany; 3 Hematology Department, Sysmex Europe SE, Bornbarch 1, 22848 Norderstedt, Germany; 4 MSH Medical School Hamburg–University of Applied Sciences and Medical University, Am Kaiserkai 1, 20457 Hamburg, Germany

**Keywords:** Infection, Sepsis, C-reactive protein (CRP), Procalcitonin (PCT), Intensive care infection score (ICIS), Cellular biomarkers

## Abstract

**Background:**

Diagnosis of infectious inflammation is challenging as acute phase protein expression is nonspecific, limiting the utility of well-established biomarkers, such as procalcitonin (PCT) and C-reactive protein (CRP). The emergent blood cell-derived Intensive Care Infection Score (ICIS) is an innovative approach for the sensitive and specific diagnosis of infection in intensive care unit (ICU) patients. This study aimed to confirm the suitability of routine ICIS use in various ICU settings.

**Methods:**

This retrospective study included 115 patients from three ICUs. Seventy-five patients were diagnosed as infected and 40 as uninfected. ICIS, CRP, and PCT were compared to routine clinical assessment to evaluate their effectiveness in predicting infection in critically ill patients.

**Results:**

ICIS was superior to CRP and PCT in discriminating infection from no infection on day 1 in the ICU. In receiver operating characteristic curve analysis, ICIS exhibited an AUC = 0.984, sensitivity of 90.7%, specificity of 97.5%, positive predictive value (PPV) of 97.7% and negative predictive value (NPV) of 89.9%, by the best cutoff value of 3. CRP gave an AUC = 0.727, PPV of 70.0% and NPV of 67.8% by best cutoff value of 8.3 mg/L with a sensitivity of 74.7% and specificity of 62.5%. The best cutoff value of 0.9 ng/mL was calculated for PCT with an AUC = 0.812, PPV of 84.4%, NPV of 70.3%, sensitivity of 69.3% and specificity of 85.0%.

**Conclusions:**

ICIS outperformed CRP and PCT in identifying infection in critically ill patients across different ICU settings on the first day in the ICU. The high NPV emphasizes the potential of ICIS as an adjuvant tool to rule out infections thereby facilitating the reduction of antibiotic overuse and consequently limiting antimicrobial resistance (AMR) development. ICIS appears suitable for routine implementation in various ICU settings.

**Graphical Abstract:**

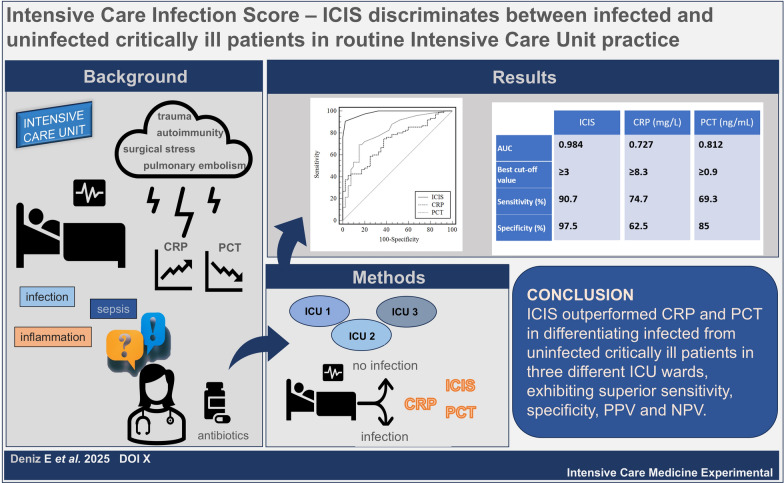

## Background

Common indicators of infection, such as fever and elevated heart rate, lack specificity as they may also be associated with other critical conditions including malignancy, autoimmunity, issues related to xenobiotics and pulmonary embolism [1–7]. Furthermore, critical illnesses within the intensive care unit (ICU) can manifest symptoms that closely resemble those of infections, including sepsis, thereby complicating the process of differentiation [8–10]. In addition, distinguishing between sepsis (suspected infection plus organ failure) and systemic inflammatory response syndrome (SIRS) presents a significant challenge, as SIRS can arise from a variety of conditions, both infectious and non-infectious, including trauma, surgical procedures, and other acute inflammatory responses [11–13]. 

The current lack of specific biomarkers to delineate these complex clinical scenarios frequently results in the unnecessary or excessive administration of antibiotics, as a precaution against overlooking a treatable infectious disease. This situation underscores the imperative need for rapid and accurate detection of infections in the ICU, along with the timely initiation of appropriate treatment to mitigate mortality rates associated with these conditions [14]. Even though multiple clinical and biochemical sepsis biomarkers have been proposed, up to now there is no single marker of sepsis that has gained general acceptance [15–17]. Despite investment in cutting-edge technologies, the discovery of disease-specific biomarkers in blood remains elusive [11, 15, 17].

C-reactive protein (CRP) and procalcitonin (PCT) are widely used biomarkers for the detection and monitoring of infections in septic patients. However, the diagnostic performance of both markers is variable. Changes in CRP and PCT levels can occur after infection and during recovery, but such changes are not exclusively due to the infection itself. In addition to infections, inflammation caused by trauma or surgical stress, which leads to tissue damage, can also affect the levels of these biomarkers [16, 18–22]. They are thus limited in their ability to distinguish infection from other non-infectious inflammatory conditions [16, 23–27].

As the technology of modern, fully automated hematology blood cell analyzers has improved significantly in recent years, other parameters of cell activation can now be measured, and quantitative cell counts of mature/immature cells can be performed. It is now possible to assess the metabolic activation of white blood cells (WBC) such as polymorphonuclear neutrophils based on the measurement of total nucleic acid and protein components in the nucleus and cytoplasm of different leukocytes [28–31]. In this context, Sysmex XN/XR-Series hematology analyzers can assess 57 cell population data (CPD) and 36 routine parameters in a blood sample [30, 32–34]. Some of those CPD parameters have already been assessed as biomarkers for bacterial infection, not limited to, but also in the context of sepsis [28, 30, 33, 35–37].

The Intensive Care Infection Score (ICIS) is currently available as licensed research use only (RUO) application within the Sysmex *Extended* Information Processing Unit (*Extended* IPU) work area manager (Sysmex Europe SE, Norderstedt, Germany) of the XN/XR hematology analyzers (Sysmex, Kobe, Japan). The score (0–20) comprises various WBC-differential, platelet, and reticulocyte-derived CPD parameters from routine blood cell counts, providing an accurate and quantitative assessment of the likelihood of infection in critically ill patients in the ICU setting [38, 39].

The ICIS parameters provide a comprehensive assessment of the early innate immune response by taking into account the following information from the differential count of white blood cells, red blood cells and platelet-related factors: the count of mature and immature granulocytes, the activation level of segmented (mature) neutrophils, the number of antibody-synthesizing lymphocytes, and the effect on hemoglobinization reflected in the difference in cellular hemoglobin equivalent between newly formed (reticulocytes) and mature red blood cells. In addition, further information from the blood count is used for algorithm validation steps to ensure a specific interpretation of the response to infection. Collectively, these parameters provide real-time information on the activity of the immune system in response to infections, enabling more accurate and prompt clinical assessments. In addition, a technical plausibility check is conducted for each measurement under the ICIS RUO license, being implemented in the *Extended* IPU, to ensure that all measurement values included in the ICIS result calculation are valid and reliable. ICIS has already been shown to aid clinicians in making timely decisions regarding the diagnosis of infections and it may offer an opportunity to monitor antibiotic treatment, leading to an improved patient outcome [38–46].

This study aimed to determine the diagnostic accuracy of ICIS in critically ill patients with infection in contrast to uninfected patients and its suitability for use in daily practice in various ICU settings by comparing it to clinical diagnosis, according to best actual practice. Furthermore, the performance of ICIS was compared with that of the established biomarkers CRP and PCT, which are widely used in hospital laboratories for diagnosing infection.

## Methods

### Study design and participants

This study was conducted retrospectively on adult patients in three ICUs at the DRK Kliniken in Berlin, Germany, between the 1st of July 2022 and the 1st of July 2023. All three ICU wards, located in different urban districts; Berlin–Westend, Berlin–Mitte and Berlin–Köpenick, admit both medical and general surgical patients, with the latter also supporting patients from the urology and hematology–oncology wards located at that hospital campus. Furthermore, as the Berlin–Köpenick Hospital serves an area of Berlin that has the highest number of elderly residents, the ICU patients here tend to be older and sicker due to the higher prevalence of comorbidities. Data from 115 patients (65 males, 50 females) were included and analyzed. Inclusion criteria were as follows: (I) an ethylenediaminetetraacetic acid (EDTA) sample on the day of ICU admission was collected and this sample was processed on the XN hematology analyzer with activated ICIS RUO licensed software; and (II) CRP and PCT were measured on the day of ICU admission. Subjects were excluded from the study if they met any of the following criteria during sample collection: hematological neoplasms, pregnancy, and anti-inflammatory/anti-neoplastic therapy. The patients were classified as either having an infection, confirmed by positive bacterial culture or positive virus polymerase chain reaction (PCR), or being uninfected, where these tests were negative. Furthermore, all patients in the infection group of this study cohort demonstrated a SOFA score ≥ 2 and therewith, fulfilled sepsis-3 guideline criteria. Confirmation of infection but no other clinical information was submitted by ICU clinicians to the study team. Therewith, further sub-stratification, including severity status, such as septic shock was not possible. Due to the retrospective design using anonymized routine clinical data, the Ethics Committee waived the need for individual informed consent.

### Sample collection and biomarker measurement

The data from the blood samples, which were routinely collected on the day of ICU admission, were utilized retrospectively for this study. S-Monovettes^®^ (SARSTEDT AG & Co.KG, Nümbrecht, Germany) with EDTA K3 potassium salt in a volume of 2.6 mL was used for hematological measurements (ICIS) and S-Monovettes^®^ with lithium heparin in a volume of 7.5 mL for the measurement of serological parameters (CRP and PCT).

ICIS values were automatically calculated by the ICIS RUO licensed software from the hematological data obtained using the XN-Series hematology analyzer (Sysmex, Kobe, Japan).

CRP was quantitatively measured on a Cobas c501 platform (Roche, Mannheim, Germany) using a latex particle intensified, two-point measurement immunoturbidimetric assay.

PCT was quantitatively measured on a Cobas e601 platform (Roche, Mannheim, Germany) using an immunological sandwich assay with a monoclonal antibody directed against PCT and chemiluminescent light emission with subsequent photometrical signal detection.

### Statistical analysis

The statistical analyses were conducted using MedCalc Software (MedCalc Software Ltd., version number 22.019, Ostend, Belgium). All tested variables were distributed non-normally (Kolmogorov–Smirnov test *p* < 0.05). Group differences were evaluated using the Wilcoxon–Mann–Whitney *U* test (two-tailed probability). Receiver operating characteristic (ROC) curve analysis was performed to assess the sensitivity, specificity, positive predictive value (PPV), negative predictive value (NPV), and area under the curve (AUC) of ICIS, CRP and PCT to distinguish between infection and no infection groups. The Youden Index was calculated to determine the optimal cutoff point at which the biomarkers provided the best balance between sensitivity and specificity [47, 48]. A *p* value of ≤ 0.05 was considered statistically significant.

## Results

### Patient characteristics

A total of 115 ICU patients with a mean age of 71.7 years (range 23–98) were enrolled in this retrospective cohort study. Baseline characteristics are shown in Table 1. For statistical analysis, 75 patients (47 males, 28 females) were assigned to the infection group, based on positive bacterial culture or positive virus PCR. All infected patients also fulfilled the sepsis-3 guideline criteria by demonstrating a SOFA score ≥ 2. 40 patients (18 males, 22 females) were assigned to the uninfected group. No statistically significant difference between the two groups was observed for age, the median length of hospitalization and mortality. Although not statistically significant, a trend for death in the infection group was present (25% of the uninfected and 30.7% of infected patients died). Bacterial infection was found in 84% and viral infection in 16% of the infection group.

**Table 1:** Baseline demographic and clinical characteristics of the study cohort

Characteristic	Overall (n = 115)	No infection (n = 40)	Infection (n = 75)	*p* value
Mean age in years	71.7 (23–98)	70.4 (23–87)	73.0 (23–98)	0.27
Length of hospitalization	19.5 (1–90)	20 (2–90)	19 (1–51)	0.77
Mortality (%)	33 (28.7%)	10 (25.0%)	23 (30.7%)	0.53
Sex				
Male	65 (56.5%)	18 (45.0%)	47 (62.7%)	
Female	50 (43.5%)	22 (55.0%)	28 (37.3%)	
Source of infection				
Bacterial		0	63 (84%)	
Viral		0	12 (16%)	

Data presented as mean (range) or as number of patients (percentage) where appropriate. A *p* value of ≤ 0.05 was considered statistically significant

### Discrimination between infected and uninfected groups

**Fig. 1:**
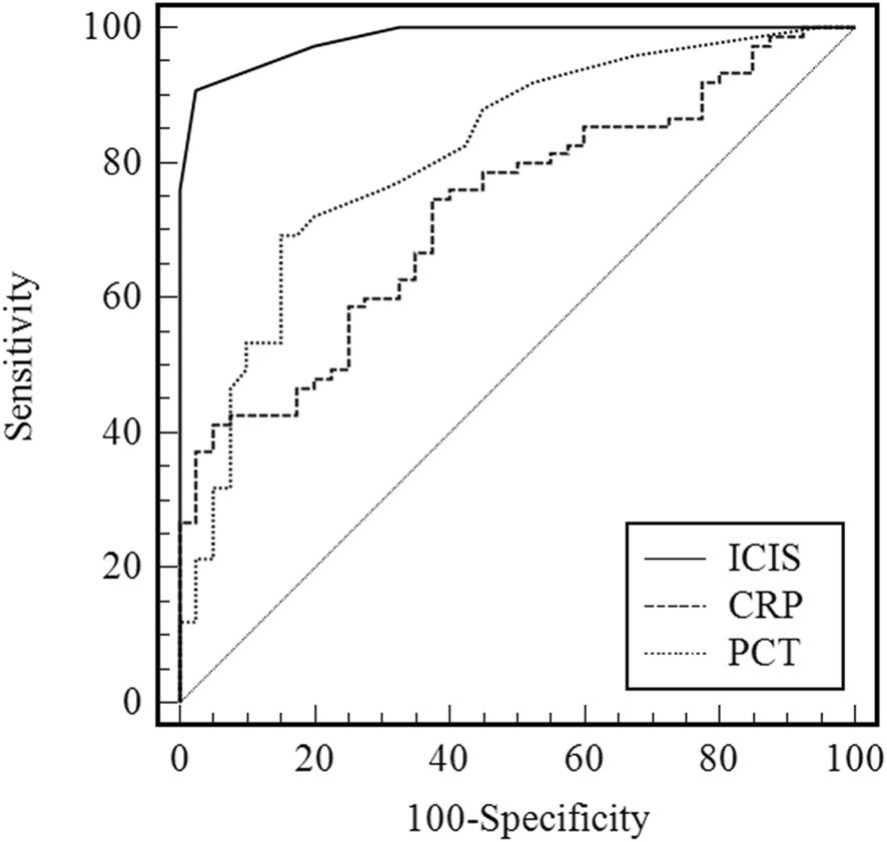
Receiver operating characteristic (ROC) curves to predict infection on the first day in ICU between intensive care infection score (ICIS), C-reactive protein (CRP) and procalcitonin (PCT)

All three biomarkers showed a statistically significant discriminatory power between the uninfected and infected group with a* p* value < 0.0001 (Fig. 1, Table 2). ICIS demonstrated a statistically significant difference towards CRP and PCT (*p* < 0.0001), whereas no statistically significant difference was observed between CRP and PCT (*p* = 0.081 data not shown).

**Table 2:** Sensitivity and specificity for detection of infection on the first day in ICU

	ICIS	CRP (mg/L)	PCT (ng/mL)
AUC	0.984	0.727	0.812
Best cutoff value	≥ 3	≥ 8.3	≥ 0.9
Sensitivity (%)	90.7	74.7	69.3
Specificity (%)	97.5	62.5	85.0
*p* value (no infection vs. infection)	< 0.0001	< 0.0001	< 0.0001
*p* value (ICIS vs. other biomarkers)	–	< 0.0001	< 0.0001

Receiver operating curve (ROC) analysis was performed to determine the area under the curve (AUC) and the best cutoff value for each variable to discriminate between uninfected and infected patients on the first day in the intensive care unit (ICU). Sensitivity and specificity are calculated from the best cutoff value

The significance of differences between ICIS and other biomarkers (CRP and PCT) is given as *p* values. A *p* value of ≤ 0.05 was considered statistically significant. Intensive Care Infection Score (ICIS); C-reactive protein (CRP); Procalcitonin (PCT)

The AUC of ROC analysis for the prediction of infection on the first day in ICU was highest for ICIS, followed by PCT and lowest for CRP (Fig. 1, Table 2). The highest sensitivity and specificity were achieved by ICIS. CRP demonstrated higher sensitivity than PCT, but lower specificity.

Considering the prevalence from the present study cohort of 65.2% for infections in ICU patients, the PPV and NPV were highest for ICIS, lower for PCT and lowest for CRP, using the best cutoff values from the present study (Table 3). After applying literature cutoff values for all three biomarkers to our data set, the PPV and NPV were highest for ICIS; PPV was lowest for PCT, whereas NPV was lowest for CRP (Table 3). Furthermore, all three biomarkers revealed higher PPV along with lower NPV when using the cutoff values from the literature.

**Table 3:** Positive and negative predictive value for infection on the first day in ICU

	*using the current study cutoffs*			*using the literature cutoffs*		
	ICIS	CRP (mg/L)	PCT (ng/mL)	ICIS	CRP (mg/L)	PCT (ng/mL)
Cutoff	≥ 3	≥ 8.3	≥ 0.9	≥ 5	≥ 20	≥ 2
Sensitivity (%)	90.7	74.7	69.3	76.0	26.7	49.3
Specificity (%)	97.5	62.5	85.0	100.0	97.5	90.0
PPV (%)	98.6	78.9	89.7	100.0	95.2	90.2
NPV (%)	84.8	56.8	59.7	69.0	41.5	48.7

Positive predictive value (PPV) and negative predictive value (NPV) are calculated from sensitivity and specificity using the following cutoff values; best cutoff from the present study; cutoff values from literature; ICIS: [40, 41]; CRP: [48]; PCT: [49]. The prevalence of 65.2% from the present study cohort was adopted. Intensive Care Unit (ICU); Intensive Care Infection Score (ICIS); C-reactive protein (CRP); Procalcitonin (PCT)

### Performance characteristics

**Fig. 2:**
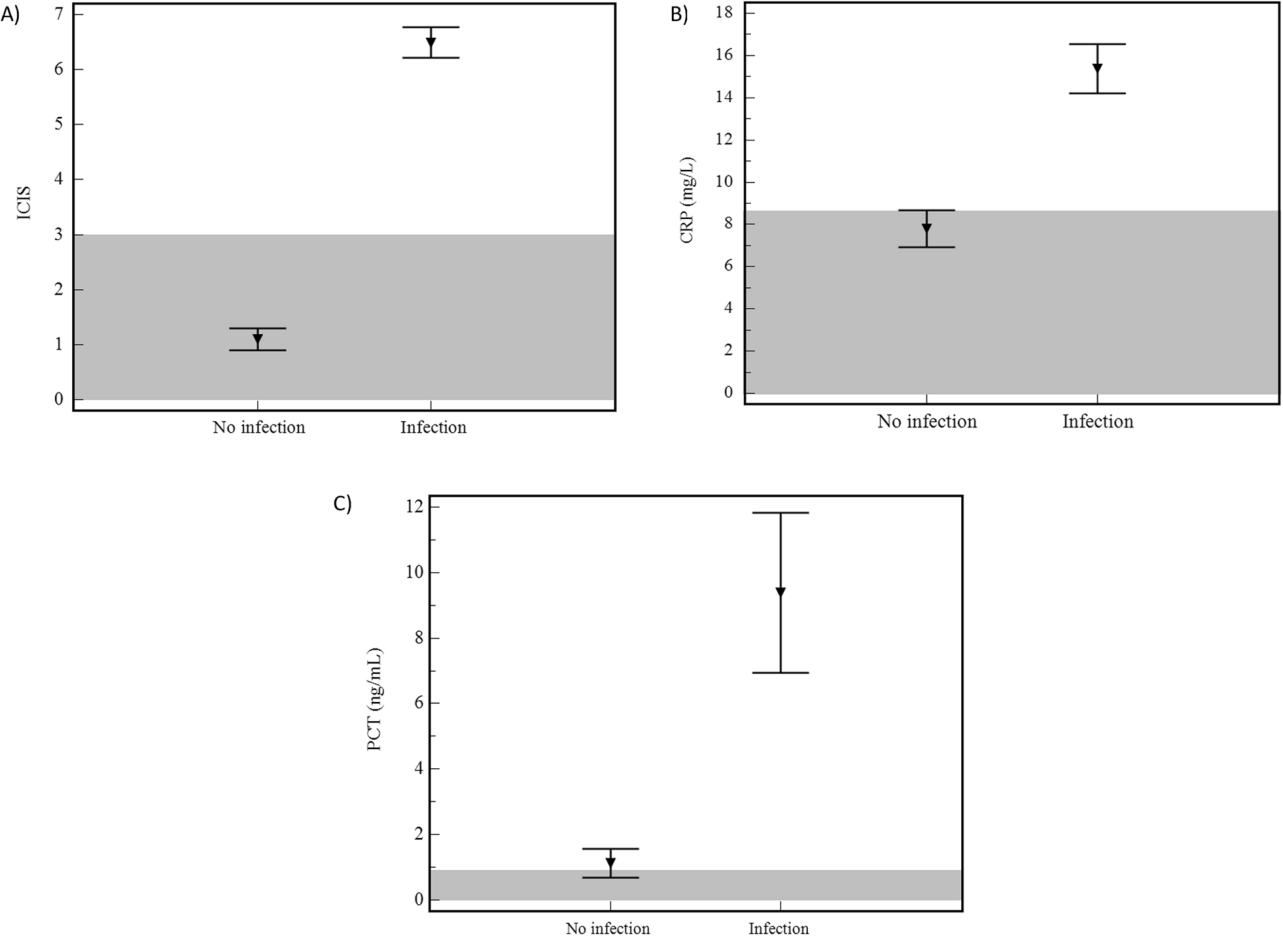
Discriminative power of ICIS, CRP and PCT between uninfected and infected patients on the first day in ICU. Means ± 1SEM (standard error of the mean) are plotted for (**A**) intensive care infection score (ICIS), (**B**) C-reactive protein (CRP) and (**C**) procalcitonin (PCT). The grey area demonstrates the range below the best cutoff values calculated from the study cohort using receiver operating curve (ROC) analysis: for ICIS < 3; CRP < 8.3 mg/L and PCT < 0.9 ng/mL

A statistically significant difference between the two evaluated patient groups was observed on the first day in ICU for all three biomarkers; ICIS, CRP and PCT, demonstrating higher values in infected compared to uninfected patients (Fig. 2A–C, Table 4).

**Table 4:** ICIS, CRP, and PCT values to distinguish uninfected and infected patients

	Mean	Lowest value	Highest value	*p* value
ICIS				
No infection	1.1	0	4	< 0.0001
Infection	6.5	2	12	
CRP				
No infection	7.8	0.1	21.6	0.0001
Infection	15.4	0.4	46.8	
PCT				
No infection	1.1	0.0	15.3	< 0.0001
Infection	9.4	0.1	100.0	

Mean, lowest and highest values as well as *p* values are indicated for each variable for the no infection and infection group. A *p* value of ≤ 0.05 was considered statistically significant

## Discussion

This retrospective study aimed to further illustrate the suitability of using ICIS in daily practice in various ICU settings. The capability of ICIS to discriminate between infected and uninfected patients was confirmed by our evaluation in three different ICU wards. Our data indicate that ICIS (cutoff ≥ 3) was significantly better than CRP and PCT in distinguishing whether a patient was infected or not on the first day in the ICU. It also demonstrated higher sensitivity and specificity (90.7% and 97.5%, respectively), along with the highest PPV and NPV.

The prevalence of infection on the first day in ICU of this study cohort was 65.2%. Whereas the prevalence of infections, in general, within the first 24 h after admission to ICU exceeds 50%, the nosocomial infections range from 15.6% [47], to 20.6% [48] up to 22% [49], including infections acquired after 24 h.

For CRP, with a PPV of 78.9%, using 8.3 mg/L as the cutoff, 21.1% of infectious events would have been missed. For PCT, using a cutoff of 0.9 ng/mL, the PPV was 89.7%, the number of potentially missed infectious events was lower (10.3%), whereas ICIS would have missed only 1.4% with a cutoff of 3 in this study cohort. On the other hand, utilizing the NPV, the potentially missed true non-infectious events were equally high for CRP and PCT, at 43.2% and 40.3%, respectively, and lowest for ICIS (15.2%). A meta-analysis of nine studies concluded that PCT cannot be utilized as rule-out criteria in critically ill patients with suspected sepsis [50]. Besides this limitation of common biomarkers, the results of this present study illustrate the potential of ICIS (demonstrating the highest rate of true negative results) as a new adjuvant tool to rule out infections and thus the possibility to reduce unnecessary antibiotic treatment and consequently, the potential to fight against antimicrobial resistance (AMR). For all three biomarkers the NPV was higher using the cutoff values of 3 for ICIS, 8.3 mg/L for CRP and 0.9 ng/mL for PCT, identified as having the best discriminatory power in this study, comparing them to the higher cutoff values adopted from literature (ICIS: 5, CRP: 20 mg/L, and PCT: 2 ng/mL) [40, 41, 51, 52]. While the performance metrics changed, the analysis still supports the potential utility of ICIS relative to CRP and PCT under those conditions, particularly noting its high PPV (100%) and reasonable NPV (69%) at the literature cutoff. As an important additional perspective on relative performance, the comparison between cutoff values derived from the present study cohort and partly very dissimilar literature cutoffs, emphasizes the demand of cutoff value evaluation for various ICU settings to be utilized properly in diagnosing infection (as one part of sepsis-3 criteria) and antibiotic treatment decision-making in critically ill patients.

Many studies have been conducted to determine the diagnostic accuracy of CRP and PCT in detecting infections and sepsis. These studies have shown that although PCT is superior to CRP, both biomarkers still show only moderate performance as judged by AUC, sensitivity and specificity, which is consistent with the results found in this study [23, 53–56]. It is also important to note that numerous recent studies have shown that CRP and PCT alone or in combination cannot accurately detect infections, regardless of the nature of the patient cohorts [14, 57–59]. This underlines the need for new and effective biomarkers that would predict infection in all types of patients in an ICU, which is what triggered the initial development of ICIS [38]. ICIS has been assessed in various patient cohorts, which demonstrated its potential for predicting infection more effectively than CRP and PCT, like our results [38–40, 46]. In these studies, different ICIS cutoff values (3 to 5) were evaluated in various patient cohorts (prospective, retrospective, post-operative, lower respiratory tract infections, amongst others). Weimann *et.al.* determined the best cutoff value for ICIS as 3, being predictive for infection, which concurs with our results [39]. Similar to our findings, in a recent study ICIS demonstrated a diagnostic performance for early infection detection at admission, with an AUC of 0.958, outperforming CRP, PCT and IL6 [46]. In that study, ICIS cutoff of ≥ 4 provided a high sensitivity and specificity (93.3% and 84.2%, respectively) [46]. Moreover, considering the high specificity of 97.5% observed in this study, an ICIS below 3 reliably ruled out an infection, which may aid in decisions regarding antibiotic treatment. Furthermore, monitoring the trend of ICIS (descending or ascending) over an ICU stay reflects the response of the immune system under treatment and might be utilized for better and more prompt antibiotic stewardship to combat further expansion of AMR [39].

Accurate detection of infections directly and indirectly impacts healthcare costs. CRP and PCT, with their moderate infection detection capacity, indirectly lead to significant costs by promoting unnecessary antibiotic use. Furthermore, the direct costs of these tests negatively impact healthcare budgets, particularly in low- and middle-income countries [60–64]. ICIS not only provides accurate detection of infection but also offers a faster and cost-effective alternative to the traditional biomarkers CRP and PCT [39, 40, 46]. ICIS detection requires only one blood tube (EDTA), typically already part of the routine blood draw without additional burden for the patient, along with a software license. In contrast, CRP and PCT measurements require an additional blood tube (serum or lithium–heparin plasma) and sampling, as well as the corresponding instruments to perform the tests. This disparity can pose challenges for patient blood management, particularly in critically ill patients. Furthermore, an immunoassay for CRP or PCT requires an analysis time of up to 30 min and a centrifugation step of 10 min, whereas ICIS measured can be completed in under 2 min. Hence, besides being cost-effective and less labor-intensive, ICIS provides time efficiency, which is crucial for decision-making in ICUs.

Nevertheless, the findings of this study must be seen in light of some limitations. Given the restricted access to the clinical information system, potential confounding factors, such as other underlying health conditions or treatments received by patients, were not considered for the comparison of biomarkers. Since ICIS provide real-time information on the immune system activity, patients with hematological neoplasms or receiving anti-inflammatory/anti-neoplastic therapy would distort the values of ICIS, hence the results of this evaluation cannot be extrapolated for the patient groups that were excluded from the study. Moreover, the findings of the present study are specific to this culture/PCR-positive cohort and the performance in patients with high clinical suspicion of infection, but negative microbiological results warrant separate investigation. As the lack of a gold standard for infectious diseases poses a common challenge in evaluating novel diagnostics, a clinically adjudicated reference may be considered as comparator method for further evaluations [65]. Future studies should also address the evaluation of ICIS performance in various non-infectious inflammatory states. Furthermore, the results reflect the first day in ICU and, therefore, do not provide any information regarding ICU-acquired infections. In addition, the relatively small sample size of the present study does not permit statistics on sub-cohorts and thus limits its generalizability alongside the retrospective study design.

Future studies could include a larger number of infected and uninfected ICU patients, providing more comprehensive information on outcomes and dynamics within these groups. The high mortality rate observed in the uninfected group (25%) might be due to late-onset infections or other complications, which is underlined by the long hospital stay of this group (20 days on average, up to 90 days in total). The utilization of ICIS for the early identification of hospital-acquired infections in critically ill patients after admission to an ICU could be evaluated with consecutive monitoring in future studies. This study serves as a validation trial to confirm the power of ICIS to discriminate between infection and no infection in critically ill patients and its suitability for use in routine clinical practice in various ICU settings.

## Conclusion

Based on our results, ICIS outperformed CRP and PCT in differentiating infected and uninfected patients in three different ICU wards, exhibiting superior sensitivity, specificity, PPV and NPV.

## Data Availability

Data are available from the authors upon reasonable request and with permission from the institutional board of the *Institute of Laboratory Medicine, DRK Kliniken Berlin Westend*.

## Abbreviations

AMR: Antimicrobial resistance
AUC: Area under the curve
CRP: C-reactive protein
EDTA: Ethylenediaminetetraacetic acid
ICIS: Intensive care infection score
ICU: Intensive care unit
IPU: Information processing unit
NPV: Negative predictive value
PCR: Polymerase chain reaction
PCT: Procalcitonin
PPV: Positive predictive value
ROC: Receiver operating characteristic
RUO: Research use only
SOFA: Sequential organ failure assessment
